# Angle stability improvement using optimised proportional integral derivative with filter controller

**DOI:** 10.1016/j.heliyon.2024.e38944

**Published:** 2024-10-05

**Authors:** Abdul Waheed Khawaja, Nor Azwan Mohamed Kamari, Muhammad Ammirrul Atiqi Mohd Zainuri, Syahirah Abd Halim, Mohd Asyraf Zulkifley, Shaheer Ansari, Abdul Sattar Malik

**Affiliations:** a Department of Electrical Engineering, Faculty of Engineering & Technology, Bahauddin Zakariya University, Multan, 60800, Pakistan; b Department of Electrical Electronic and Systems Engineering, Faculty of Engineering and Built Environment, Universiti Kebangsaan Malaysia, Bangi, 43000, Selangor, Malaysia; c Electric Mobility and Inteligent Vehicle Technologies, Centre for Automotive Research (CAR), Faculty of Engineering & Built Environment, Universiti Kebangsaan Malaysia, Bangi, 43000, Malaysia; d Department of Engineering, School of Engineering and Technology, Sunway University, Petaling Jaya, 47500, Selangor, Malaysia

## Abstract

Load inconsistency has disrupted the power system, causing rotor angle fluctuation that leads to angle instability in the system. This research suggests an innovative proportional integral derivative with filter (PIDF)-based thyristor-controlled series compensator (TCSC) controller that utilise an evolutionary programming sine cosine algorithm (EPSCA) for hybrid optimisation to increase the angle stability of the power system. The challenge of the PIDF-TCSC design is transformed into an optimal control problem with respect to performance indices, such as the maximum imaginary part of system eigenvalues, damping ratio and damping factor, where another multi-objective function is utilised to determine the best stabiliser settings. Eigenvalue analysis is used to conduct the stability study in a linearised paradigm of the single-machine infinite-bus (SMIB) network. The resilience of the PIDF controller was tested using a SMIB power network under various operating circumstances. Simulation results are used to evaluate the system's effectiveness with the proposed optimised PIDF-TCSC controller to that of the system using the proportional integral derivative, proportional integral, and base case PIDF-TCSC approaches. The research findings demonstrate the efficacy of EPSCA in implementing PIDF-TCSC motif and its excellent resilient performance for improving power system stability as related to other strategies in various situations.

## 1 Introduction

To establish a reliable large-interconnected power system, synchronous machines should synchronize with other electrical systems such as generators whilst maintaining a synchronous state. A system perturbation results in low-frequency oscillations, which affect electrical generation, transmission and distribution. This phenomenon could cause a machine to become unstable, trip other components and cause the system to fail [[1], [2], [3], [4]]. Various models and modelling methodologies, including coordination control, flexible alternating current transmission systems and power system stabilisers, have been utilised for damping. In such cases, FACTS are the best choice to damp the inter-area modes of oscillations. The controller design of FACTS devices determines their damping performance [5]. A high-performance and affordable series FACTS device is the thyristor-controlled series capacitor (TCSC), which is frequently used in real-world power systems for precise and secure optimal power flow regulation in power lines [6,7]. One of the most cost-effective ways to free up the size of power lines to transport additional active power is through the series of compensation provided by TCSC [8]. By adjusting the tie-series line's impedance with variable TCSC reactance dynamically, the reduction of tie-line power and inter-area low-frequency oscillations becomes feasible [9]. The coordination control scheme provides effective damping in the local and inter-area modes of oscillation. However, a lack of coordination amongst the controllers can cause system instability because of their interaction. Therefore, the design of damping controllers is a huge task to interact properly in large systems damp low-frequency frequency oscillations effectively [5].

A fundamental component of the stability of a single-machine infinite bus (SMIB) power system is the development of damping controllers. The design challenges include the selection of controller types and their appropriate parameters [[10], [11]]. In SMIB systems and multi-machine, the controller design is very complex because of number parameter optimisation problems. For stability study, the SMIB system, which is based on eigenvalue analysis, is modelled linearly. The optimisation of damping controllers in a linearised model of the SMIB system is linked with the selection of controller, formulation of an objective function and selection of optimisation technique [5,12].

In recent years, many types of FACTS devices have been used to eliminate low-frequency oscillations in electrical network by constructing controllers that absorb or infuse reactive energy. In this case, static VAR compensators (SVC) and static synchronous compensators (STATCOM) [13], were employed to improve the angle stability [14,15]. The best position to use the SVC in the transmission lines is at midsection or at the transmitting end, coupled with on buses and branches. However, PV buses are not permitted to have SVC [16]. For wind power facilities, STATCOM provides the stability margin for transients and a unified power factor. Additionally, the authors discuss the improvement of low-voltage ride-through capability in power systems, which is a dynamic control technique to boost power flow capabilities [17], and a damping performance analysis unified power flow controller (UPFC) [18]. The UPFC is a versatile unit that regulates the flow of the reactive and active energies along a line, the magnitude of the bus voltage, the impedance of power lines and the phase angle [16]. To reduce local and inter-mode oscillations in power systems, FACTS and PSS units have been incorporated using coordination control through efficient controller coordination. The thyristor-controlled series capacitor (TCSC), SVC and PSS coordination control concepts were disclosed in Refs. [[19], [20], [21]]. TCSC is a reactive power injection and variable reactance model with an ideal reactive power injection. It cannot be used at branches with a transformer [16]. Furthermore, PSS has been coordinated with STATCOM, UPFC and IPFC [[22], [23], [24]].

One of the most affordable ways to allow transmission lines to carry more active power is through series compensation, which is provided by TCSC [7,8,25,26]. A complementary power swing damping controller is used in TCSC to enhance the multi-machine power systems' rotor angle stability [27]. The implementation of a power swing damping controller and jointly-operating power system stabilisers (PSS) to boost the small-signal stability of power systems seems to have become widespread in recent years (PID and lead-lag) [[28], [29], [30], [31], [32]]. The TCSC and TCPS are thyristor-controlled switches, whereas the SSSC is a voltage-source converter that depends on gate turn off (GTO) switches. The SSSC is a costly solution, and it is more complicated and expensive than the TCSC [33,34]. The TCSC is more affordable than the SSSC because capacitors are less expensive than GTOs. However, the TCSC has a significantly greater practical foundation than the more expensive and advanced SSSC, which lacks a standalone in-service practical application 1′ [[34], [35], [36]]. By adjusting the tie-series line's impedance with variable TCSC reactance dynamically, the reduction of tie-line power and area frequency oscillations become feasible. With time delay concerns, a TCSC is situated in a two-area thermal–thermal system's series with the tie-line. In collaboration with TCSC, SMES units are installed in both locations to improve system dynamism. For TCSC, a progressive model that is comparable to the approach suggested in Refs. [9,37,38] has already been created.

A number of publications investigated lead-lag, proportional integral derivative (PID) and proportional-integral (PI) controllers whilst developing the objective function for the SMIB system's stability study [10,[39]], [40], [41], [42], [42]a], [43], [44], [45], [46]]. In multi-phase flow behaviour patterns in pipelines and risers at seaward gushers, the proportional integral derivative with filter (PIDF) finds its place [47], management of load frequency in an unregulated electricity system [48], managing reactive power for the power system's transient and steady-state stability [49], industrial processes to obtain genuine resolution for the prominent process oscillation [50], and buck converter for DC–DC converters [51]. The efficiency of the hybrid microgrid system was improved by optimizing the fuzzy PIDF load frequency controller based on the marine predator algorithm [45].

Various controllers have been developed by implementing different indictors which infuse or absorb reactive energy to reduce oscillations in electrical network in the past years. These indicators include the damping factor, damping ratio and maximum eigenvalue. The multi-objective function has a mathematical expression that is associated with two or more aims in the optimisation process for designing a damping controller. In recent research, the worth of multi-objective functions is observed to attain formulation in optimisation by various research for design damping controller parameters [46,[52], [53], [54], [55], [56], [57]]. Locating eigenvalues in the D-shaped region of complex s-plane by the formulation of multi-objective technique in controller design is preferred. Various methods have been used to place eigenvalues in the D-shape region of the s-plane [52,[54], [55], [56]]. The formulation D-shaped technique is debated for dominant modes based on damping ratio and damping factor [56,58,59]. This technique considers some selected modes for optimisation. The algebraic sum of the damping factor and damping ratio, which create the D-shaped geographical area of the s-plane and enable it to have less damping, is another method for establishing the multi-objective function [53]. The formulation objective function in damping controller design is relatively complex, and its relative performance must be assessed under a common threshold. The damping ratio indicator reduces oscillation overshoots. However, it performs poorly in terms of settling time. Similar finding was obtained for the damping factor used as indicator. Hence, the single indicator cannot offer greater accuracy in the areas of settling time and oscillatory maximum overshoot.

In the last decades, many optimisation algorithms have been developed to optimise controller parameters for damping schemes. The optimisation techniques are conventional, deterministic, heuristic and hybrid. The conventional method uses the concept of control theory to optimise the constraints of the controller in the frequency domain. The damping controller parameters are optimised by using classical optimisation techniques [5]. Classical methods have a problem of control schemes not effectively working when their control is not readjusted according to the system response. This problem is solved by modern control theory-based algorithms to design damping schemes [5,60]. However, several complications of modern theory-based techniques have been reported [52]. The complications include the order reduction, selection weighting function, and phenomena of pole-zero cancellation in it. Deterministic techniques forecast the upcoming actions from previous sets of actions and their use to optimise controller parameters [61]. Sequential quadratic programming was used to design a controller for the best damping performance [39]. In these algorithms, the selection of the initial point is critical, and finding an optimum solution is extremely difficult. In the case of a large interconnected power system, solution convergence is not possible because of the large number of parameters that need to be optimised. Heuristic optimisation techniques are globally popular procedures for finding the optimum solution by a stochastic method. In this method, solutions are found by trial and error. Metaheuristic techniques are the upgraded form of heuristic techniques. Most of these techniques are nature-inspired algorithms that do not require the prediction of initial solutions. The heuristic techniques provide flexible and effective robust optimisation for damping controller designs [5].

Genetic algorithm (GA) is a metaheuristic algorithm that was anticipated to design a multimachine system and formulate the multi-objective function for damping controller [[62], [62]a],^63^]. GA was used in the coordination design of PSS and TCSC [64,65]. The disadvantage of GA is the premature convergence problem that is due to local minima stagnation issues [5]. Evolutionary programming (EP) is a heuristic method performed by employing an innate method to obtain the elite result in the search space, an EP-based technique was presented to improve the voltage profile in a radial distribution system [66] and its use to calculate a generator's normal operating transient and sub-transient characteristics [67]. EP method has a local minima stagnation problem, and its final solution is influenced by some parameters and their choice [5,58,59]. A population-based metaheuristic optimisation algorithm called particle swarm optimisation (PSO) is inspired by nature. PSO may solve multimodal optimisation problems to design the damping controllers of SMIB and multimachine systems [[68], [69], [70]]. PSO has a local minima stagnation problem, and its final solution is influenced by some parameters and their choice [5]. The moth flame optimisation (MFO) technique is a metaheuristic algorithm. MFO was expected to improve voltage stability through the formulation of multi-objective function and optimal power flow control in power systems [71]. The best placement for STATCOM and its parameter settings was selected using the MFO method [72]. The MFO algorithm was used to mitigate low-frequency oscillation using a multi-machine power system to develop the stabiliser [73]. MFO's drawbacks include delayed convergence and trapping in local optima [74]. The sine cosine algorithm (SCA) has several advantages over other heuristic techniques. The performance of SCA in the comparative analysis of multimodal optimisation problems is remarkable [75]. SCA is a stochastic population-based optimisation metaheuristic method that draws inspiration from nature and may easily be escaped from the local minima trap and no problem of premature convergence [58,59, 75]. The SCA technique, however, converges the solution in a tad bit more iterations [76]. To certify the effective and robust design of the damping controllers, some variations in SCA are required before its implementation to boost the damping performance of the power system. The summary of the accomplished work-based angle stability improvement with suitable controller is presented in Table 1.

**Table 1:** Comparative analysis of some literature work for angle stability.

References	Methods	Advantages	Disadvantage
5,13,14,15	VAR compensators and Static compensators	VAR compensators (SVC) and STATCOM were employed to improve the angle stability.	It is not possible to offer actual damping against every local area oscillation mode.
17	UPFC	Provide improvement of low-voltage ride-through capability in power systems	Complex circuitry and its control
19–21	FACTS and PSS	FACTS and PSS units have been incorporated using coordination control through efficient controller coordination	Improperly coordinated design of controllers may affect system stability by negative damping.
49	PID	Controllers whilst developing the objective function for the SMIB system's stability study.	Constraint in finding of eigenvalue location in the complicated s-plane's D-shaped region.
5,54,64-65	GA	To design a system and formulate the multi-objective function for damping controller.	GA has a local minima stagnation problem.
5,62–63.	PSO	Able to resolve complex optimisation issues across several application fields.	PSO has a local minima stagnation problem, and the parameters chosen will affect the solution in the end.
5,58–59,66	EP	Easy to learn and implement without much knowledge.	Premature convergence.
71–74	MFO	Offers comparatively satisfactory solutions for some optimisation concerns.	Slow convergence rate.

The following are this paper's main contributions:

⁃ A damping controller was developed using a new mathematical model of a PIDF controller and its power stability feature.
⁃ A reliable damping controller for a SMIB platform was built using the evolutionary programming SCA (EPSCA) technique.
⁃ A revolutionary multi-objective (MO) function that was created and includes a potent damping controller design for SMIB systems can be utilised in large electrical systems.
⁃ The traditional PID and PI methodologies are managed to validate the proposed EPSCA-based PIDF controller.

Section 2 describes how to model a TCSC-SMIB power system and a TCSC-SMIB system using PIDF, PID, PI and PI controllers. This section also proposes a multi objective function. Section 3 explains the suggested EPSCA algorithm. Section 4 presents the results of three separate loading condition simulations for comparison. Lastly, Section 5 provides a summary of the findings.

## 2 Materials and methods

FACTS is now a common, efficient and cost-effective tool used in lengthy transmission lines to eliminate oscillations in the power system. The source of inter-area oscillations in a large interconnected power system is the lack of a generation plant near a tie line and lengthy power lines. To dampen inter-area oscillations in huge, integrated power networks adequately, a FACTS-based stabiliser is used as an alternative.

### 2.1 Modelling of a SMIB power system

**Fig. 1:**
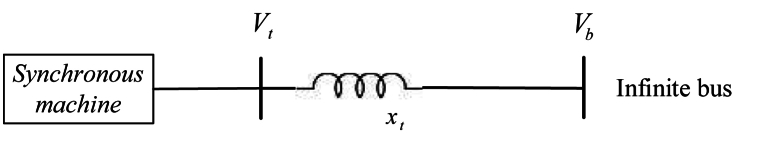
Power system with one machine and infinite bus [2].

Fig. 1 depicts a power system with a generator that supplies power to a large network. The large network's capacity is significantly greater than that of the generator. Thus, modifications to the area of the network to the left of busbar *b* (Fig. 1) have no impact whatsoever on the operation of the large network. This finding effectively means that when the study's attention is on the section of network on the left, that is, at busbar b, the voltage and frequency remain constant. Therefore, the large network's capacity is ‘infinite’ from the perspective of how the left-hand portion of the network operates. As a result, busbar *b* is referred to as the ‘infinite busbar’, and the network component to its left is referred to as a ‘single-machine infinite-bus’ power system. The SMIB power network simulates a real power system where power lines connect a power plant with a generator or set of generators to a large power network. Fig. 1 depicts a synchronous machine connected to an infinite bus with voltage $V_{b}$ through transmission lines with impedance $x_{t}$ and an excitation system with terminal voltage $V_{t}$ [2].

The resistance of transmission line is supposed to be zero ($R_{e}=0)$ . The SMIB power system is depicted simply in Fig. 1:

(1) $$V_{t}=jx_{t}I_{t}+V_{b}$$

Equation (1) can be expressed in the d–q coordinate of the generator as follows:

(2) $$v_{td}+jv_{tq}=jx_{t}\left(i_{d}+ji_{q}\right)+v_{d}+jv_{q}\text{,}$$

where the *d* and *q* components of the generator's terminal voltage, $V_{t}$, the line current, $I_{t}$, and the voltage at the infinite busbar, $V_{b}$, are denoted by $v_{td}$, $v_{tq}$, and $i_{d}$, $i_{q}$, $v_{d}$, and $v_{q}$, respectively. The real and imaginary parts on the two sides of Equation (4.2) can be compared and yield

(3) $$v_{td}=-x_{t}i_{q}+v_{d}\text{,}$$

(4) $$v_{tq}=x_{t}i_{d}+v_{q}\text{,}$$

where $v_{d}=V_{b}\;\sin\;\delta$, and $v_{q}=V_{b}\;\cos\;\delta$.

(5) $$V_{t}=\sqrt{v_{td}+v_{tq}}\text{.}$$

The well-known Park's voltage equations serve as the foundational equations for characterising the dynamics of a synchronous generator. They are provided using a coordinate system with a direct axis (*d*-axis) placed on the field winding axis of a synchronous generator and a quadrature axis (*q*-axis). The synchronous generator's stator's armature phase windings (e.g. a, b and c) are transformed into their corresponding armature phase windings (e.g. *d* and *q*) by Park's transformation. The *Q* and *D* damper windings of the rotor are persistently short-circuited. Field winding *f* energises direct current. The simplest form can be found in Park's voltage calculations for individuals windings as follows:

(6) $${\dot{\Psi }}_{d}=\omega_{o}\left(v_{td}+r_{a}i_{d}+\omega \Psi_{q}\right)\text{,}$$

(7) $${\dot{\Psi }}_{q}=\omega_{o}\left(v_{tq}+r_{a}i_{q}-\omega \Psi_{d}\right)\text{,}$$

(8) $${\dot{\Psi }}_{f}=\omega_{o}\left(v_{f}-r_{f}i_{f}\right)\text{,}$$

(9) $${\dot{\Psi }}_{D}=-\omega_{o}r_{D}i_{D}\text{,}$$

(10) $${\dot{\Psi }}_{Q}=-\omega_{o}r_{Q}i_{Q}\text{,}$$

where, for the synchronous generator, $\omega_{o}$ indicates the reference speed, and ω signifies the rotor speed. The magnetic flux linkage, current, voltage and resistance of each respective winding are represented by $\Psi_{d}$, $\Psi_{q}$, $\Psi_{f}$, $\Psi_{D}$, $\Psi_{Q}$, $i_{d}$, $i_{q}\text{,}$ $i_{f}\text{,}$ $i_{D}$, $i_{Q}$, $v_{td}$, $v_{tq}$, $v_{f}$, $r_{a}\text{,}$ $r_{f}$, $r_{D}$ and $r_{Q}$.

The generator's simplest excitation system has a mathematical model that is expressed as follows:

(11) $$E_{fd}=E_{fdo}+E_{fd}^{\prime }\text{,}$$

(12) $${\dot{E^{\prime }}}_{fd}=-\frac{E_{fd}^{\prime }}{T_{A}}+\frac{K_{A}}{T_{A}}\left(V_{tref}-V_{t}+u_{stab}\right)\text{,}$$

where $V_{t}$ , $V_{tref}$, $K_{A}$*,* and $T_{A}$ are the generator's terminal voltage, reference setting value, AVR gain and AVR time constant, respectively; and $E_{fdo}$ is the persistent excitation. The stabilising signal is represented by $u_{stab}$.

The synchronous generator's rotor motion equation is expressed as follows:

(13) $$\dot{\delta }=\omega \left(\omega_{o}-1\right)\text{,}$$

(14) $$\dot{\omega }=\frac{1}{M}\left(T_{m}-T_{e}-D\left(\omega -1\right)\right)\text{,}$$

where δ signifies the angle of the synchronous generator's rotor with regard to a base axis; *M* signifies the rotor's inertia; *D* denotes the rotor motion's damping coefficient; and $T_{m}$ and $T_{e}$ symbolise the mechanical torque and electric torque applied to the generator's rotor, respectively.

The per unit mechanical and electric torques are also equal ($P_{m}=T_{m},\text{and}\;P_{t}=T_{e}$) when the generator's output of electric power is equal to the prime mover's input of mechanical power. $P_{t}$ is equal to the expected quantity of electricity at the infinite busbar. Hence,

(15) $$P_{t}=v_{td}i_{d}+v_{tq}i_{q}=v_{d}i_{d}+v_{q}i_{q}\text{.}$$

The SMIB power system's basic dynamic model is depicted in Fig. 1 and is represented by Equations (3), (4), (5), (6)–(10), (11)–(12), (13)–(14) and (15). $V_{b}$ and $P_{m}$ are both constant.

Based on the following criteria, the intricate mathematical formulation of the synchronous machines used in Equation (6), (7), (8), (9), (10) for the research of power system oscillations can be simplified:

The impact of damper windings not considered in Equation (6), (7), (8), (9), (10) is reduced to

(16) $${\dot{\Psi }}_{d}=\omega_{o}\left(v_{td}+r_{a}i_{d}+\omega \Psi_{q}\right)\text{,}$$

(17) $${\dot{\Psi }}_{q}=\omega_{o}(\left(v_{tq}+r_{a}i_{q}-\omega \Psi_{d}\right)\text{,}$$

(18) $${\dot{\Psi }}_{f}=\omega_{o}\left(v_{f}-r_{f}i_{f}\right)\text{.}$$

The effect of rapid pulses is disregarded along with the resistance of the *d* and *q* armature windings. The simplified version of Equations (16), (17), (18) are

(19) $$0=\omega_{o}\left(v_{td}+\omega \Psi_{q}\right)\text{,}$$

(20) $$0{=\omega }_{o}\left(v_{tq}-\omega \Psi_{d}\right)\text{,}$$

(21) $${\dot{\Psi }}_{f}=\omega_{o}\left(v_{f}-r_{f}i_{f}\right)\text{.}$$

The fluctuation of rotor speed in small-signal power oscillations is very small. Consequently, Equations (16), (17) become

(22) $$v_{td}=-\Psi_{q}\text{,}$$

(23) $$v_{tq}=\Psi_{d}\;\text{.}$$

It is defined to change Equation (21) into a different form.

(24) $$E_{q}^{\prime }=\frac{x_{ad}}{x_{f}}\Psi_{f},E_{q}=x_{ad}i_{f},E_{fd}=\frac{x_{ad}}{r_{f}}v_{f}$$

where $E_{q}^{\prime }$ q-axis transitory excitation voltage, $E_{q}$ q-axis transitory excitation voltage and $E_{fd}$ excitation voltage are used interchangeably. $x_{ad}$ and $x_{aq}$ refer to mutual inductances. The two sides of Equation (21) are multiplied by $\frac{x_{ad}}{r_{f}}$, thereby yielding

(25) $$T_{do}^{\prime }{\dot{E^{\prime }}}_{q}=E_{fd}-E_{q}\text{,}$$

where the field winding's time constant, $T_{do}^{\prime }$, equals $\frac{x_{f}}{r_{f}\omega_{o}}$. $x_{f}$ refers to the reactance of direct current excited winding *f*. The third-order simplified synchronous generator model is formed using Equations (25), (13), (13), (14). From Equations (22), (23), (24), we yield

(26) $$v_{td}=x_{q}i_{q}\text{,}$$

(27) $$v_{tq}=E_{q}-x_{d}i_{d}\text{,}$$

(28) $$E_{q}^{\prime }=E_{q}-x_{d}^{\prime }i_{d}\text{,}$$

(29) $$T_{do}^{\prime }{\dot{E^{\prime }}}_{q}=E_{fd}-E_{q}^{\prime }-\left({{x_{d}-x}^{\prime }}_{d}\right)i_{d}\text{,}$$

where the transient *d*-axis reactance, denoted by $x_{d}^{\prime }=x_{d}-\frac{{x_{ad}}^{2}}{x_{f}}$, exists. According to Equations (3), (4), (27), Fig. 1 illustrates that a SMIB power system can have,

(30) $$v_{td}=x_{q}i_{q}$$

(31) $$v_{tq}=E_{q}^{\prime }-x_{d}^{\prime }i_{d}$$

Thus,

(32) $$v_{d}=\left({x_{t}+x}_{q}\right)i_{q}=x_{q\Sigma }i_{q}$$

(33) $$v_{q}=E_{q}^{\prime }-\left(x_{d}^{\prime }+x_{t}\right)i_{d}=E_{q}^{\prime }-x_{d\Sigma }^{\prime }i_{d}=E_{q}-x_{d\Sigma }i_{d}$$

The SMIB power system can be illustrated using the system on the d–q axis. From Equation (33), we yield

(34) $$i_{d}=\frac{E_{q}-V_{b}\;\cos\;\delta }{x_{d\Sigma }^{\prime }}\text{,}$$

(35) $$i_{q}=\frac{V_{b}\;\sin\;\delta \;}{x_{q\Sigma }}\text{.}$$

By changing Equations (3), (4), (34), (34), (35) into Eq. (15), the electrical energy produced by the generator can be stated as follows:

$$P_{t}=V_{b}\;\cos\;\delta \frac{V_{b}\;\sin\;\delta }{x_{q\Sigma }}+V_{b}\;\sin\;\delta \frac{E_{q}^{\prime }-V_{b}\;\cos\;\delta }{x_{d\Sigma }^{\prime }}$$

(36) $$P_{t}=\frac{V_{b}\;E_{q}^{\prime }}{x_{d\Sigma }^{\prime }}\sin\;\delta -\frac{{V_{b}}^{2}\left(x_{q}-x_{d}^{\prime }\right)}{2x_{q\Sigma }x_{d\Sigma }^{\prime }}\sin\;2\;\delta \text{.}$$

From Equations (29), (34)–(35),

(37) $$E_{q}=\frac{E_{q}^{\prime }x_{d\Sigma }}{x_{d\Sigma }^{\prime }}-\frac{\left(x_{d}-x_{d}^{\prime }\right)}{x_{d\Sigma }^{\prime }}V_{b}\;\cos\;\delta$$

where $x_{d\Sigma }=x_{d}+x_{t}$. From Equations (26), (27), (28), (34)–(35),

(38) $$v_{td}=\frac{x_{q}V_{b}\;\sin\;\delta }{x_{q\Sigma }}\text{,}$$

(39) $$v_{tq}=\frac{x_{t}E_{q}^{\prime }}{x_{d\Sigma }^{\prime }}+\frac{x_{d}^{\prime }}{x_{d\Sigma }^{\prime }}V_{b}\;\cos\;\delta$$

Consequently, Equations ((5) and (12)–(14) and (25) and (36)–(39) represent the simpler version of the SMIB power system.

By linearising Equations ((5) and (12)–(14) and (25) and (36)–(39), the electricity system is operationally located with parameters $\delta =\delta_{o}$, $\omega_{o}=1$, $E_{q}^{\prime }=E_{qo}^{\prime }$, $E_{fd}=E_{fdo}$, $V_{t}=V_{to}$, $v_{td}=v_{tdo}$, $v_{tq}=v_{tqo}$. Thus, it can be obtained that

(40) $$\Delta \dot{\delta }=\omega_{o}\Delta \omega \text{,}$$

(41) $$\Delta \dot{\omega =\frac{1}{M}\left(-\Delta P_{t}-D\Delta \omega \right)}$$

(42) $$\Delta {{\dot{E}}^{\prime }}_{q}=\frac{1}{{T^{\prime }}_{do}}\left(-\Delta E_{q}+\Delta {E^{\prime }}_{fd}\right)$$

(43) $$\Delta {{\dot{E}}^{\prime }}_{fd}=-\frac{1}{T_{A}}\Delta E_{fd}^{\prime }-\frac{K_{A}}{T_{A}}\left(\Delta V_{t}-\Delta u_{stab}\right)\text{,}$$

(44) $$\Delta P_{t}=K_{1}\Delta \delta +K_{2}\Delta E_{q}^{\prime }\text{,}$$

(45) $$\Delta E_{q}=K_{4}\Delta \delta +K_{3}\Delta E_{q}^{\prime }$$

(46) $$\Delta V_{t}=K_{5}\Delta \delta +K_{6}\Delta E_{q}^{\prime }$$

where,

(47) $$K_{1}=\frac{E_{qo}^{\prime }V_{b}}{x_{d\Sigma }^{\prime }}\cos\;\delta_{o}-\frac{{V_{b}}^{2}\left(x_{q}-x_{d}^{\prime }\right)}{x_{q\Sigma }x_{d\Sigma }^{\prime }}\cos\;{2\delta }_{o}\text{,}$$

(48) $$K_{2}=\frac{V_{b}\;}{x_{d\Sigma }^{\prime }}\sin\;\delta_{o}\text{,}$$

(49) $$K_{3}=\frac{x_{d\Sigma }}{x_{d\Sigma }^{\prime }}\text{,}$$

(50) $$K_{4}=\left(x_{d}-x_{d}^{\prime }\right)\frac{V_{b}\;}{x_{d\Sigma }^{\prime }}\sin\;\delta_{o}\text{,}$$

(51) $$K_{5}=\frac{v_{tdo}}{V_{to}}\frac{x_{q}V_{b}\;\cos\;\delta_{o}}{x_{q\Sigma }}-\frac{v_{tqo}}{V_{to}}\frac{x_{d}^{\prime }}{x_{d\Sigma }^{\prime }}V_{b}\;\sin\;\delta_{o}\text{,}$$

(52) $$K_{6}=\frac{v_{tqo}}{V_{to}}\frac{x_{t}}{x_{d\Sigma }^{\prime }}\text{,}$$

where, $K_{1}$, $K_{2}$, $K_{3}$, $K_{4}$, $K_{5}$ and $K_{6}$ are constants, and their values as equations (47), (48), (49), (50), (51), (52). Substituting equations (44), (45), (46) into equations (40), (41), (42), (43), it can be obtained that,

(53) $$\Delta \dot{\omega }=\frac{1}{M}\left(-K_{1}\Delta \delta {-K}_{2}\Delta E_{q}^{\prime }-D\Delta \omega \right)$$

(54) $$\Delta {\dot{E^{\prime }}}_{q}=\frac{1}{T_{do}^{\prime }}\left(-K_{3}\Delta E_{q}^{\prime }{-K}_{4}\Delta \delta +\Delta E_{fd}^{\prime }\right)$$

(55) $$\Delta {\dot{E^{\prime }}}_{fd}=-\frac{1}{T_{A}}\Delta E_{fd}^{\prime }-\frac{K_{A}}{T_{A}}\left(K_{5}\Delta \delta +K_{6}\Delta E_{q}^{\prime }-\Delta u_{stab}\right)$$

**Fig. 2:**
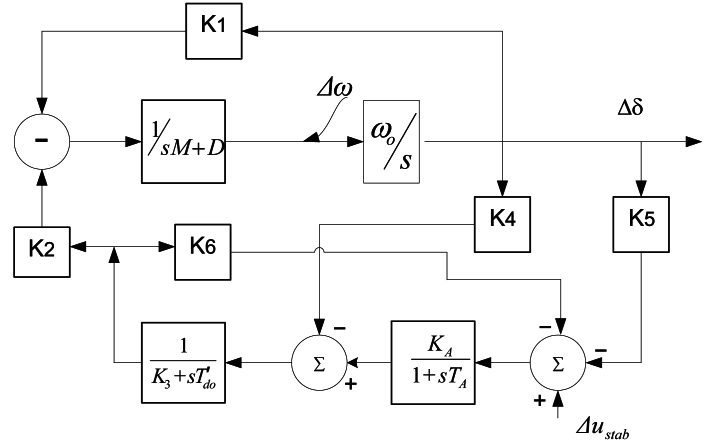
SMIB power system according to the Heffron-Phillips paradigm [2].

Equations (40), (53)– (55) serve as a representation of the Heffron–Phillips paradigm of a SMIB power system and is depicted in Fig. 2 [2].

### 2.2 Modelling of a TCSC-SMIB power system

**Fig. 3:**
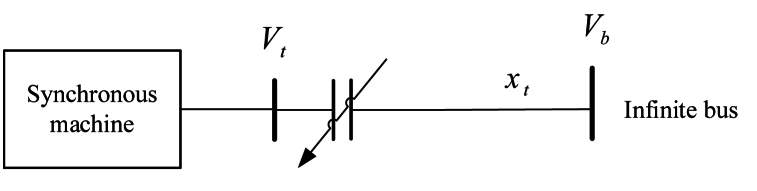
TCSC equipped with a single-machine infinite-bus electrical network [76].

The SMIB energy infrastructure with a TCSC, utilised to stabilization is illustrated in Fig. 3. Fig. 3 shows a synchronous machine including a synchronous machine-based excitation system, terminal voltage $V_{t}$, a TCSC damping controller and power lines with reactance $x_{t}$ attached to an infinite bus with voltage $V_{b}$. $x_{tcsc}$ is an acronym for TCSC variable reactance. It is used to design TCSC damping controllers [76].

Equations (13), (14), (25), (12), (56)–(61) represent the simplified paradigm of SMIB electrical network with a TCSC.

(56) $$P_{t}=\frac{E_{q\;}^{\prime }V_{b}}{\left(x_{d}^{\prime }+x_{t}-x_{tcsc}\right)}\sin\;\delta -\frac{V_{b}^{2}}{2}\left(x_{q}-x_{d}^{\prime }\right)\frac{\sin\;2\;\delta }{\left(x_{d}^{\prime }+x_{t}-x_{tcsc}\right)\left(x_{q}+x_{L}-x_{tcsc}\right)}\text{,}$$

(57) $$E_{q}=\frac{\left(x_{d}+x_{L}-x_{tcsc}\right)E_{q\;}^{\prime }}{\left(x_{d}^{\prime }+x_{t}-x_{tcsc}\right)}-\frac{\left(x_{d}-x_{d}^{\prime }\right)V_{b}\;\cos\;\delta }{\left(x_{d}^{\prime }+x_{t}-x_{tcsc}\right)}\text{,}$$

(58) $$E_{fd}=E_{fdo}+E_{fd}^{\prime }\text{,}$$

(59) $$v_{td}=\frac{x_{q}V_{b}\;\sin\;\delta }{\left(x_{q}+x_{L}-x_{tcsc}\right)}\text{,}$$

(60) $$v_{tq}=\frac{x_{d}^{\prime }V_{b}\;\cos\;\delta }{\left(x_{d}^{\prime }+x_{t}-x_{tcsc}\right)}+\frac{E_{q\;}^{\prime }x_{t}}{\left(x_{d}^{\prime }+x_{t}-x_{tcsc}\right)}\text{,}$$

(61) $$V_{t}=\sqrt{v_{td}^{2}+v_{tq}^{2}}\text{.}$$

By linearising Equations (13), (14), (25), (12), (56) at an electricity system operational point, where $\delta =\delta_{o}$, $\omega_{o}=1$, $E_{q}^{\prime }=E_{qo}^{\prime }$, $E_{fd}=E_{fdo}$, $V_{t}=V_{to}$, $v_{td}=v_{tdo}$, $v_{tq}=v_{tqo}$, it can be obtained that,

We have

(62) $${\Delta P}_{t}=K_{1}^{\prime }\Delta \delta +K_{2}^{\prime }E_{q\;}^{\prime }+K_{p}{\Delta x}_{tcsc}\text{,}$$

(63) $${\Delta E}_{q}=K_{4}^{\prime }\Delta \delta +K_{3}^{\prime }E_{q\;}^{\prime }+K_{q}{\Delta x}_{tcsc}\text{,}$$

(64) $${\Delta V}_{t}=K_{5}^{\prime }\Delta \delta +K_{6}^{\prime }E_{q\;}^{\prime }+{\Delta x}_{tcsc}\text{,}$$

where

(65) $$K_{1}^{\prime }=\frac{E_{qo\;}^{\prime }V_{b}\;\cos\;\delta_{o}}{\left(x_{d}^{\prime }+x_{t}-x_{tcsc}\right)}-\frac{V_{b}^{2}\left(x_{q}-x_{d}^{\prime }\right)\cos\;2\delta_{o}}{\left(x_{d}^{\prime }+x_{t}-x_{tcsc}\right)\left(x_{q}+x_{L}-x_{tcsc}\right)}\text{,}$$

(66) $$K_{2}^{\prime }=\frac{V_{b}\;\sin\;\delta_{o}}{\left(x_{d}^{\prime }+x_{t}-x_{tcsc}\right)}\text{,}$$

(67) $$K_{3}^{\prime }=\frac{\left(x_{d}+x_{L}-x_{tcsc}\right)}{\left(x_{d}^{\prime }+x_{t}-x_{tcsc}\right)}\text{,}$$

(68) $$K_{4}^{\prime }=\left(x_{d}-x_{d}^{\prime }\right)K_{2}^{\prime }$$

(65) $$K_{1}^{\prime }=\frac{E_{qo\;}^{\prime }V_{b}\;\cos\;\delta_{o}}{\left(x_{d}^{\prime }+x_{t}-x_{tcsc}\right)}-\frac{V_{b}^{2}\left(x_{q}-x_{d}^{\prime }\right)\cos\;2\delta_{o}}{\left(x_{d}^{\prime }+x_{t}-x_{tcsc}\right)\left(x_{q}+x_{L}-x_{tcsc}\right)}\text{,}$$

(66) $$K_{2}^{\prime }=\frac{V_{b}\;\sin\;\delta_{o}}{\left(x_{d}^{\prime }+x_{t}-x_{tcsc}\right)}\text{,}$$

(67) $$K_{3}^{\prime }=\frac{\left(x_{d}+x_{L}-x_{tcsc}\right)}{\left(x_{d}^{\prime }+x_{t}-x_{tcsc}\right)}\text{,}$$

(68) $$K_{4}^{\prime }=\left(x_{d}-x_{d}^{\prime }\right)K_{2}^{\prime }$$

(69) $$K_{5}^{\prime }=\frac{V_{b}\;\cos\;\delta_{o}x_{q}v_{tdo}}{V_{to}\left(x_{q}+x_{L}-x_{tcsc}\right)}-\frac{v_{tqo}{{x^{\prime }}_{d}V}_{b}\;\sin\;\delta_{o}}{V_{to}\left(x_{d}^{\prime }+x_{t}-x_{tcsc}\right)}\text{,}$$

(70) $$K_{6}^{\prime }=\frac{v_{tqo}\left(x_{L}-x_{tcsc}\right)}{V_{to}\left(x_{d}^{\prime }+x_{t}-x_{tcsc}\right)}$$

(71) $$K_{p}=\frac{E_{qo\;}^{\prime }V_{b}\;\sin\;\delta_{o}}{{\left({x^{\prime }}_{d}+x_{t}-x_{tcsc}\right)}^{2}}-\frac{V_{b}^{2}\left(x_{q}-x_{d}^{\prime }\right)\left(x_{d}^{\prime }+x_{t}-x_{tcsc}\right)+\left(x_{q}+x_{L}-x_{tcsc}\right))\;\sin\;{2\delta }_{o}}{2{\left({x^{\prime }}_{d}+x_{t}-x_{tcsc}\right)}^{2}\left(x_{q}+x_{L}-x_{tcsc}\right){\;}^{2}}\text{,}$$

(72) $$K_{q}=\frac{E_{qo\;}^{\prime }\left(x_{d}+x_{L}-x_{tcsc}\right)}{{\left({x^{\prime }}_{d}+x_{t}-x_{tcsc}\right)}^{2}}-\frac{\left(x_{d}-x_{d}^{\prime }\right)V_{b}\;\cos\;\delta_{o}}{{\left({x^{\prime }}_{d}+x_{t}-x_{tcsc}\right)}^{2}}\text{,}$$

(73) $$K_{v}=\frac{{v_{tdo}x_{q}V}_{b}\;\sin\;\delta_{o}}{V_{to}\left(x_{q}+x_{L}-x_{tcsc}\right){\;}^{2}}+\frac{v_{tqo}}{V_{to}}\left[\left(\frac{x_{L}}{{\left({x^{\prime }}_{d}+x_{t}-x_{tcsc}\right)}^{2}}-\frac{1}{\left(x_{d}^{\prime }+x_{t}-x_{tcsc}\right)}\right)E_{qo\;}^{\prime }+\frac{x_{d}^{\prime }V_{b}\;\cos\;\delta_{o}}{{\left({x^{\prime }}_{d}+x_{t}-x_{tcsc}\right)}^{2}}\right]\text{,}$$

where $K_{1}^{\prime }$, $K_{2}^{\prime }$, $K_{3}^{\prime }$, $K_{4}^{\prime }$, $K_{5}^{\prime }$, $K_{6}^{\prime }$, $K_{p}$, $K_{q}$ and $K_{v}$ are constants for TCSC with SMIB system, and their values are expressed as Equations (65)–(73). Substituting Equations (62)–(64) into equations (12)–(14) and (25), it can be obtained that,

(74) $$\Delta \dot{\omega }=\frac{1}{M}\left(-K_{1}^{\prime }\Delta \delta -D\Delta \omega -K_{2}^{\prime }\Delta E_{q\;}^{\prime }-K_{p}\Delta x_{tcsc}\right)\text{,}$$

(75) $${\Delta \dot{E^{\prime }}}_{q}=\frac{1}{T_{do}^{\prime }}\left(-K_{4}^{\prime }\Delta \delta -K_{3}^{\prime }\Delta E_{q\;}^{\prime }-K_{q}\Delta x_{tcsc}+\Delta E_{fd}^{\prime }\right)\text{,}$$

(76) $$\Delta {\dot{E^{\prime }}}_{fd}=-\frac{{\Delta E}_{fd}^{\prime }}{T_{A}}-\frac{K_{A}}{T_{A}}\left(K_{5}^{\prime }\Delta \delta +K_{6}^{\prime }\Delta E_{q\;}^{\prime }+K_{v}\Delta x_{tcsc}\right)\text{,}$$

**Fig. 4:**
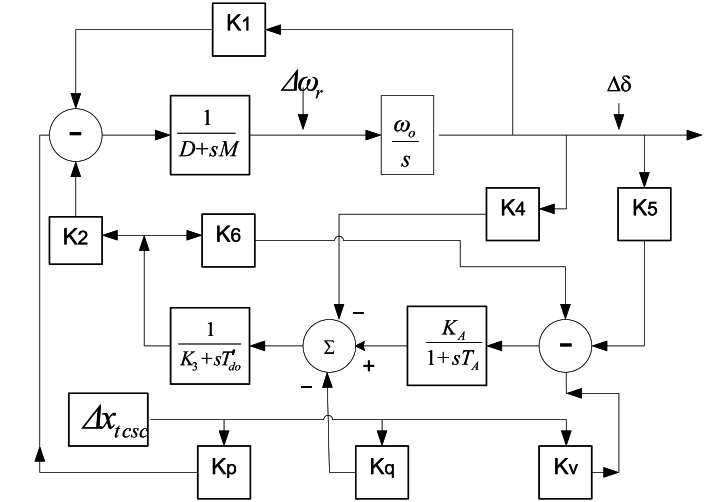
SMIB power system with a TCSC stabiliser incorporated, according to an upgraded Heffron-Phillips paradigm [2].

Hence, Equations (40), (74) are the upgraded Heffron–Phillips paradigm of the SMIB electrical network fixed with the TCSC stabiliser (Fig. 4) [2]. A linearised state space mathematical model (LSSMM) is used to express the system in Fig. 4 as follows:

(77) $${\dot{X}}_{1}=G_{tcsc}.X_{1}+H_{tcsc}.I$$

(78) $${\dot{X}}_{1}=\left[\Delta \dot{\delta }\Delta \dot{\omega }\Delta {\dot{E^{\prime }}}_{q}\Delta {\dot{E^{\prime }}}_{fd}\right]$$

(79) $$X_{1}=\left[\Delta \delta \Delta \omega \Delta E_{q\;}^{\prime }\Delta E_{fd}^{\prime }\right]$$

(80) $$G_{tcsc}=\left[\begin{array}{cccc} 0 & \omega_{o} & 0 & 0\\ -\frac{K_{1}^{\prime }}{M} & -\frac{D}{M} & -\frac{K_{2}^{\prime }}{M} & 0\\ -\frac{K_{4}^{\prime }}{T_{do}^{\prime }} & 0 & -\frac{K_{3}^{\prime }}{T_{do}^{\prime }} & \frac{1}{T_{do}^{\prime }}\\ -\frac{K_{A}K_{5}^{\prime }}{T_{A}} & 0 & -\frac{K_{A}K_{6}^{\prime }}{T_{A}} & -\frac{1}{T_{A}} \end{array}\right]$$

(81) $$H_{tcsc}=\left[\begin{array}{c} 0\\ -\frac{K_{p}}{M}\\ -\frac{K_{q}}{T_{do}^{\prime }}\\ -\frac{K_{A}K_{v}}{T_{A}} \end{array}\right]$$

(82) $$I=\left[\Delta x_{tcsc}\right]$$

### 2.3 TCSC SMIB system modelling with PI controller

**Fig. 5:**
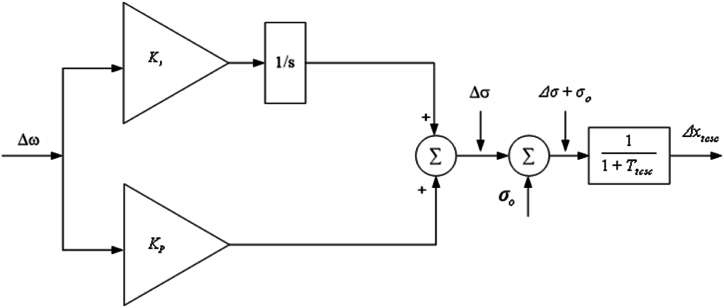
Controller schematic representation for the TCSC PI.

The image below (Fig. 5) shows the TCSC PI controller. The PI controller is represented by the following equation.

(83) $$\Delta \sigma =\left(K_{P}+\frac{K_{I}}{s}\right)\Delta \omega \text{,}$$

where $K_{P}$ and $K_{I}$ are proportional gain constant and integral gain constant of PI controller, respectively. Substituting Equation (74) into Equation (83), it can be obtained that,

(84) $$\Delta \dot{\sigma }=-\frac{K_{P}K_{1}^{\prime }}{M}\Delta \delta +\left(K_{I}-\frac{K_{P}K_{1}^{\prime }}{M}\right)\Delta \omega -\frac{K_{P}K_{2}^{\prime }}{M}{\Delta E^{\prime }}_{q\;}-\frac{K_{P}K_{p}}{M}{\Delta x}_{tcsc}\text{,}$$

(85) $$\Delta {\dot{x}}_{tcsc}=\frac{1}{T_{tcsc}}\Delta \sigma -\frac{1}{T_{tcsc}}\Delta x_{tcsc}\;\text{.}$$

Hence, Equations (40) and (74)–(76) and (84) – (85) are the upgraded Heffron–Phillips paradigm of the SMIB electrical network fixed with PI controller TCSC stabiliser. A LSSMM TCSC with PI controller is used to express the system in Fig. 4 and is shown as follows:

(86) $${\dot{X}}_{2}=G_{PI}.X_{2}+H_{PI}.I_{PI}\text{,}$$

(87) $$\dot{{\dot{X}}_{2}}=\left[\Delta \dot{\delta }\Delta \dot{\omega \;}{\Delta \dot{E^{\prime }}}_{q}\Delta {\dot{E^{\prime }}}_{fd}\;\Delta \dot{\sigma }\Delta {\dot{x}}_{tcsc}\right]\text{,}$$

(88) $$X_{2}=\left[\Delta \delta \Delta \omega \Delta {\overset{´}{E}}_{q}\Delta E_{fd}^{\prime }\Delta \sigma \Delta x_{tcsc}\right]\text{,}$$

(89) $$G_{PI}=\left[\begin{array}{cccccc} 0 & \omega_{o} & 0 & 0 & 0 & 0\\ -\frac{K_{1}^{\prime }}{M} & -\frac{D}{M} & -\frac{K_{2}^{\prime }}{M} & 0 & 0 & -\frac{K_{p}}{M}\\ -\frac{K_{4}^{\prime }}{T_{do}^{\prime }} & 0 & -\frac{K_{3}^{\prime }}{T_{do}^{\prime }} & \frac{1}{T_{do}^{\prime }} & 0 & -\frac{K_{q}}{T_{do}^{\prime }}\\ -\frac{K_{A}K_{5}^{\prime }}{T_{A}} & 0 & -\frac{K_{A}K_{6}^{\prime }}{T_{A}} & -\frac{1}{T_{A}} & 0 & -\frac{K_{A}K_{v}}{T_{A}}\\ -\frac{K_{1}^{\prime }K_{P}}{M} & K_{I}-\frac{K_{P}.D}{M} & -\frac{K_{2}^{\prime }K_{P}}{M} & 0 & 0 & -\frac{K_{p}K_{P}}{M}\\ 0 & 0 & 0 & 0 & \frac{1}{T_{t\;\csc}} & -\frac{1}{T_{t\;\csc}} \end{array}\right]\text{,}$$

(90) $$H_{PI}=\left[\begin{array}{c} 0\\ \frac{1}{M}\\ 0\\ 0\\ 0\\ 0 \end{array}\right]\text{,}$$

(91) $$I_{PI}=\left[\Delta T_{m}\right]\text{.}$$

### 2.4 TCSC SMIB system modelling with PID controller

**Fig. 6:**
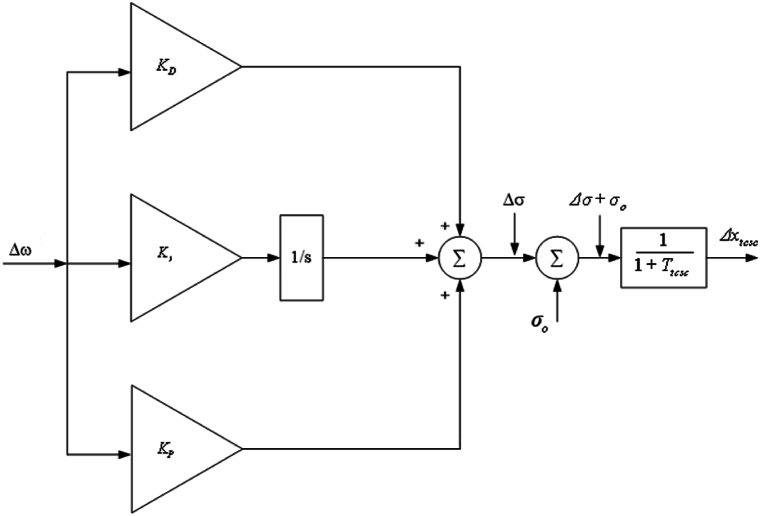
Controller schematic representation for the TCSC PID.

TCSC PID controller is as shown in Fig. 6. The PID is represented by the following equation.

(92) $$\Delta \sigma =\left(K_{P}+\frac{K_{I}}{s}+K_{D}\frac{du}{dt}\right)\Delta \omega \text{,}$$

where $K_{D}$ refers to the derivative gain constant of PID controller. Substituting Equation (74) in Equation (92), we obtain

(93) $$\Delta \dot{\sigma }=A_{51}\Delta \delta +A_{52}\Delta \omega +A_{53}\Delta E_{q\;}^{\prime }+A_{54}\Delta E_{fd}^{\prime }+A_{55}\Delta \sigma +A_{56}\Delta x_{tcsc}\text{,}$$

(94) $$\Delta {\dot{x}}_{tcsc}=\frac{1}{T_{tcsc}}\Delta \sigma -\frac{1}{T_{tcsc}}\Delta x_{tcsc}\text{,}$$

where,

(95) $$A_{51}=\frac{DK_{1}K_{D}}{M^{2}}-\frac{K_{1}K_{P}}{M}+\frac{K_{2}K_{4}K_{D}}{MT_{do}^{\prime }}\text{,}$$

(96) $$A_{52}=K_{I}-\frac{\omega_{o}K_{1}K_{D}}{M}+\frac{D^{2}K_{D}}{M^{2}}-\frac{D.K_{D}}{M}\text{,}$$

(97) $$A_{53}=\frac{DK_{2}K_{D}}{M^{2}}-\frac{K_{2}K_{P}}{M}+\frac{K_{2}K_{3}K_{D}}{M\overset{´}{T_{do}}}\text{,}$$

(98) $$A_{54}=-\frac{K_{2}K_{D}}{MT_{do}^{\prime }}\text{,}$$

(99) $$A_{55}=-\frac{K_{p}K_{D}}{MT_{tcsc}}\text{,}$$

(100) $$A_{56}=\frac{DK_{p}K_{D}}{M^{2}}-\frac{K_{p}K_{P}}{M}+\frac{K_{2}K_{q}K_{D}}{M\overset{´}{T_{do}}}+\frac{K_{p}K_{D}}{MT_{tcsc}}\text{.}$$

Hence, Equations (40) and (74)–(76) and (93) – (94) are the upgraded Heffron–Phillips paradigm of the SMIB electrical network fixed with PID controller TCSC stabiliser. A LSSMM for TCSC with PID controller is used to express the system in Fig. 4 and is shown as follows:

(101) $${\dot{X}}_{3}=G_{PID}.X_{3}+H_{PID}.I_{PID}$$

(102) $${\dot{X}}_{3}=\left[\Delta \dot{\delta }\Delta \dot{\omega \;}{\Delta \dot{E^{\prime }}}_{q}\Delta {\dot{E^{\prime }}}_{fd}\Delta \dot{\sigma }\Delta {\dot{x}}_{tcsc}\right]$$

(103) $$X_{3}=\left[\Delta \delta \Delta \omega \Delta E_{q\;}^{\prime }\Delta E_{fd}^{\prime }\Delta \sigma \Delta x_{tcsc}\right]$$

(104) $$G_{PID}=\left[\begin{array}{cccccc} 0 & \omega_{o} & 0 & 0 & 0 & 0\\ -\frac{K_{1}^{\prime }}{M} & -\frac{D}{M} & -\frac{K_{2}^{\prime }}{M} & 0 & 0 & -\frac{K_{p}}{M}\\ -\frac{K_{4}^{\prime }}{T_{do}^{\prime }} & 0 & -\frac{K_{3}^{\prime }}{T_{do}^{\prime }} & \frac{1}{T_{do}^{\prime }} & 0 & -\frac{K_{q}}{T_{do}^{\prime }}\\ -\frac{K_{A}K_{5}^{\prime }}{T_{A}} & 0 & -\frac{K_{A}K_{6}^{\prime }}{T_{A}} & -\frac{1}{T_{A}} & 0 & -\frac{K_{A}K_{v}}{T_{A}}\\ A_{51} & A_{52} & A_{53} & A_{54} & A_{55} & A_{56}\\ 0 & 0 & 0 & 0 & \frac{1}{T_{t\;\csc}} & -\frac{1}{T_{t\;\csc}} \end{array}\right]$$

(105) $$H_{PID}=\left[\begin{array}{c} 0\\ \frac{1}{M}\\ 0\\ 0\\ 0\\ 0 \end{array}\right]$$

(106) $$I_{PID}=\left[\Delta T_{m}\right]$$

### 2.5 TCSC SMIB system modelling with PIDF controller

**Fig. 7:**
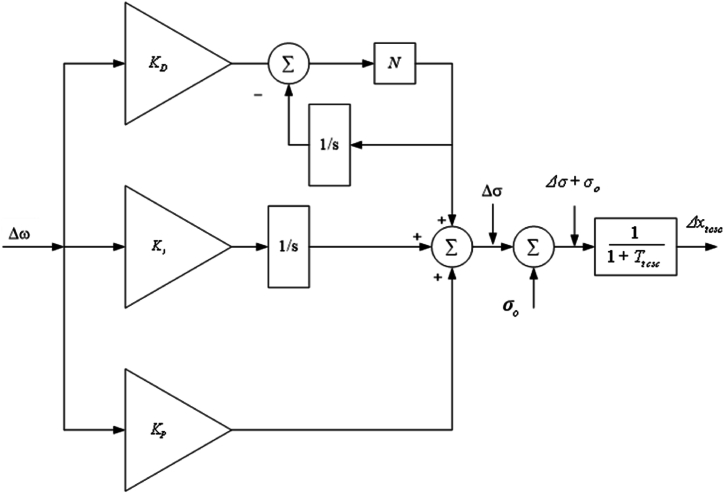
Controller schematic representation for the TCSC PIDF [76].

The following is the mathematical equation of complex frequency response of the PIDF controller with the $K_{P}$, $K_{D}$, $K_{I}$, and *N* parameters shown in Fig. 7:

(107) $$\Delta \sigma =\left(K_{P}+\frac{K_{I}}{s}+K_{D}\frac{Ns}{s+N}\right)\Delta \omega \text{,}$$

where *N* refers to derivative filter coefficient of PIDF controller. Equation (107) can be changed to have, by substituting Equation (74).

(108) $$\Delta \dot{\sigma }=\Delta f\text{,}$$

(109) $$\Delta \dot{f}=G\Delta \delta +H\Delta \omega +I\Delta E_{q\;}^{\prime }+J{\Delta E}_{fd}^{\prime }-L\Delta \sigma -N\Delta f+K\Delta x_{tcsc}\text{,}$$

(110) $$\Delta {\dot{x}}_{tcsc}=\frac{1}{T_{tcsc}}\Delta f-\frac{1}{T_{tcsc}}\Delta x_{tcsc}\text{,}$$

where,

$$G=\left(\frac{K_{P}+NK_{D}}{M}\right)\left(\frac{K_{1}D}{M}+\frac{K_{2}K_{4}}{\overset{´}{T_{do}}}\right)-\left(\frac{{NK}_{1}K_{P}+K_{1}K_{I}}{M}\right)\text{,}$$

$$H=\left(\frac{K_{P}+NK_{D}}{M}\right)\left(\frac{D^{2}}{M}-K_{1}\omega_{o}\right)-D\left(\frac{K_{I}+NK_{P}}{M}\right)+NK_{I}\text{,}$$

$$I=\left(\frac{K_{P}+NK_{D}}{M}\right)\left(\frac{K_{2}D}{M}+\frac{K_{2}K_{3}}{\overset{´}{T_{do}}}\right)-K_{2}\left(\frac{K_{I}+NK_{P}}{M}\right)\text{,}$$

$$J=K_{2}\left(\frac{K_{I}+NK_{P}}{M}\right)\text{,}$$

$$K=\left(\frac{K_{P}+NK_{D}}{M}\right)\left(\frac{K_{p}D}{M}+\frac{K_{2}K_{q}}{\overset{´}{T_{do}}}\right)-K_{p}\left(\frac{K_{I}+NK_{P}}{M}\right)+K_{p}\left(\frac{K_{P}+NK_{D}}{MT_{tcsc}}\right)\text{,}$$

$$L=-\left(\frac{K_{P}+NK_{D}}{M}\right)\left(\frac{K_{p}}{T_{tcsc}}\right)\text{,}$$

Hence, Equations (40) and (74)–(76) and (108) – (110) are the upgraded Heffron–Phillips paradigm of the SMIB electrical network fixed with PIDF controller TCSC stabiliser [76].

The system in Fig. 4 expressed by a LSSMM for TCSC PIDF controller is represented as follows:

(111) $${\dot{X}}_{4}=G_{PIDF}.X_{4}+H_{PIDF}.I_{PIDF}\text{,}$$

(112) $${\dot{X}}_{4}=\left[\Delta \dot{\delta }\Delta \dot{\omega }{\Delta \dot{E^{\prime }}}_{q}{\dot{\Delta E^{\prime }}}_{fd}\Delta \dot{\sigma }\Delta \dot{f}\Delta {\dot{x}}_{tcsc}\right]\text{,}$$

(113) $$X_{4}=\left[\Delta \delta \Delta \omega \Delta E_{q\;}^{\prime }\Delta E_{fd}^{\prime }\Delta \sigma \Delta f\Delta x_{tcsc}\right]\text{,}$$

(114) $$G_{PIDF}=\left[\begin{array}{ccccccc} 0 & \omega_{o} & 0 & 0 & 0 & 0 & 0\\ -\frac{K_{1}^{\prime }}{M} & -\frac{D}{M} & -\frac{K_{2}^{\prime }}{M} & 0 & 0 & 0 & -\frac{K_{p}}{M}\\ -\frac{K_{4}^{\prime }}{T_{do}^{\prime }} & 0 & -\frac{K_{3}^{\prime }}{T_{do}^{\prime }} & \frac{1}{T_{do}^{\prime }} & 0 & 0 & -\frac{K_{q}}{T_{do}^{\prime }}\\ -\frac{K_{A}K_{5}^{\prime }}{T_{A}} & 0 & -\frac{K_{A}K_{6}^{\prime }}{T_{A}} & -\frac{1}{T_{A}} & 0 & 0 & -\frac{K_{A}K_{v}}{T_{A}}\\ 0 & 0 & 0 & 0 & 0 & 1 & 0\\ G & H & I & J & L & -N & K\\ 0 & 0 & 0 & 0 & 0 & \frac{1}{T_{t\;\csc}} & -\frac{1}{T_{t\;\csc}} \end{array}\right]\text{,}$$

(115) $$H_{PIDF}=\left[\begin{array}{c} 0\\ \frac{1}{M}\\ 0\\ 0\\ 0\\ 0\\ 0 \end{array}\right]\text{,}$$

(116) $$I_{PIDF}=\left[\Delta T_{m}\right]\;\text{.}$$

### 2.6 Objective function

In this study, the use of a multi-objective function that provides the damping factor and damping ratio the same weight is suggested. The damping factor, damping ratio and maximum imaginary part of the system eigenvalues that create the D-shaped region in the s-plane are added algebraically to create the multi-objective function.

(117) $$Objective\;function=c_{1}.\;\frac{1}{1+|\omega_{\max}|}+c_{2}.\zeta_{\min}+c_{3}.\;\frac{\sigma_{\max}}{\sigma_{\min}}\text{,}$$

where $\left|\omega_{\max}\right|$ denotes a modulus of the determined imaginary fragment of the system eigenvalues, and $\zeta_{\min}$ expresses the minimal damping ratio; and $\sigma_{\min}$ and $\sigma_{\max}$ are the minimum and maximum damping factors, respectively. $c_{1}$. = 0.5, $c_{2}$ = 0.1, and $c_{3}$ = 0.4. The parameters $c_{1}$, $c_{2}$ and $c_{3}$ are chosen in this case according to a preliminary test that revealed that chosen amounts are excellent indicators of power system's stability state [76].

### 2.7 Evolutionary programming SCA

Abortive convergence because of the nearby least stagnating phenomenon is a disadvantage of evolutionary-based algorithms, such as evolutionary programming (EP). EP performs better throughout the exploitation exercise in contrast to the exploration exercise. The EP optimisation approach has a modest degree of convergence and is unable to deal with multimodal challenges. A population-based method called the SCA commence with a collection of random values and runs in two stages, exploration and exploitation. When implemented to investigate and utilise the maxima and minima of eigenvalue analysis, the SCA optimisation method generates favourable performance. However, the SCA method adapts the result after a several more iterations. Hence, the EP algorithm is quick but unreliable. SCA requires some time to complete, yet it is a precise procedure. Better precise data will be generated through the integration of EP and SCA. A hybrid method called the evolutionary programming SCA (EPSCA) incorporates the advantages of sine cosine and evolutionary programming algorithms. By changing the SCA technique's current features, the idea of mutation has been realised. The SCA algorithm for this idea adds three additional phases. A random number between zero and one, $u_{5}$, should be updated in the first phase. The updated and best SCA solutions are modified using two ways in the second phase: an arbitrary number or levy flight. The third stage consists of the combination of the solutions from the clone solutions and the post-mutated candidate. The algorithm then modifies the answer for each candidate. Using optimal solutions, the best option is chosen, and in the last phase, half of the choices are selected [76]. For both phases, the shifting positions are depicted by that of the following expression:

(118) $$Q_{i,j}^{v+1}=Q_{i,v}+u_{1}^{v}\times \sin\;u_{2,j}\times \left|u_{3,j}S_{d,j}^{v}-Q_{i,j}^{v}\right|\text{,}$$

(119) $$Q_{i,j}^{v+1}=Q_{j,v}+u_{1}^{v}\times \cos\;u_{2,j}\times \left|u_{3,j}S_{d,j}^{v}-Q_{i,j}^{v}\right|\text{.}$$

The following application is made by combining Equations (118), (119).

(120) $$Q_{i,j}^{v+1}=\left\{ \begin{array}{c} Q_{i,v}+u_{1}^{v}\times \sin\;u_{2,j}\times \left|u_{3,j}S_{d,j}^{v}-Q_{i,j}^{v}\right|,u_{4,j}\;<0.5\\ Q_{j,v}+u_{1}^{v}\times \cos\;u_{2,j}\times \left|u_{3,j}S_{d,j}^{v}-Q_{i,j}^{v}\right|,u_{4,j}\geq 0.5 \end{array}\right.\text{,}$$

where *v* denotes the present iteration; $S_{d,j}^{v}$ denotes the target location in the *j*-th dimension; $Q_{i,u}$ denotes the existing result at the *v*-th iteration; and $u_{1}^{v}$, $u_{2,j}$ and $u_{3,j}$ are random integers. || displays an absolute value, and $u_{4,j}$ is a random number between 0 and 1.

According to the equations above, SCA has four primary parameters: $u_{1}^{v}$, $u_{2,j}$, $u_{3,j}$ and $u_{4,j}$. The parameter $u_{1}^{v}$ determines the subsequent deployment locations (or moving pattern), which can be either within or outside the space in between result and the target. How far the movement should travel from the target in either direction is determined by the $u_{2,j}$ input. To accentuate or deemphasise the character of desalination in establishing the space stochastically, the value of the parameter $u_{3,j}$ produces random weights for the destination. The sine and cosine components of Equation (120) are then alternated equally by the parameter $u_{4,j}$.

Equation (121), which has the following form, enables the sine and cosine lengths to be changed to locate a desirable area in a given area and accelerate it to the global optima. The SCA approach performs equilibrium throughout exploitation and exploration in the following ways:

(121) $$u_{1}^{v}=c-v\frac{c}{V}\text{,}$$

where *c* is a scalar quantity, *v* is the latest iteration, and *V* is the number of iterations that can be performed. A non-Gaussian random allocation called the levy model described governs the length of its stages for long flight. Levy flight seems to be a variant of the stochastic process in Brownian motion. The following mathematical formula can be used to describe the levy distribution:

(122) $$L_{f}\left(y,r,q\right)=\left\{ \begin{array}{c} \sqrt{\frac{r}{2\pi }}\exp\left(\frac{-r}{2\left(y-q\right)}\right)\frac{1}{{\left(y-q\right)}^{\frac{3}{2}}},0˂q˂\infty \text{and}\;0˂y˂\infty \\ 0,y\leq 0\; \end{array}\right.\text{,}$$

where *y* denotes the levy distribution domain, *r* regulates the scale of the distribution, and *q* controls the location. The following explains how the standard deviation of the offspring is expressed:

(123) $$\zeta_{k}=\forall \frac{R\left(y_{k}\right)}{R_{\max}}\left(h_{l}^{\max}-h_{l}^{\min}\right)\text{,}$$

where each parent $y_{k}$ gives birth to a single offspring, $g_{k+m}$. Gaussian random variable *M* (0, $\zeta_{1}^{2}$) perturbs each optimised parameter $h_{l}$. The fitness equation $R(g_{k}$) has a trial solution, $g_{k}$, and ∀ a scaling factor. The fitness function's maximum value is represented by $R_{\max}$. The following definition applies to the offspring $g_{k+m}$:

(124) $$g_{k+m}=y_{k}+\left\{M\left(0,\zeta_{1}^{2}\right),\ldots ,M\left(0,\zeta_{n}^{2}\right)\right\}\text{,}$$

where *k* = 1, …, *m*.

**Fig. 8:**
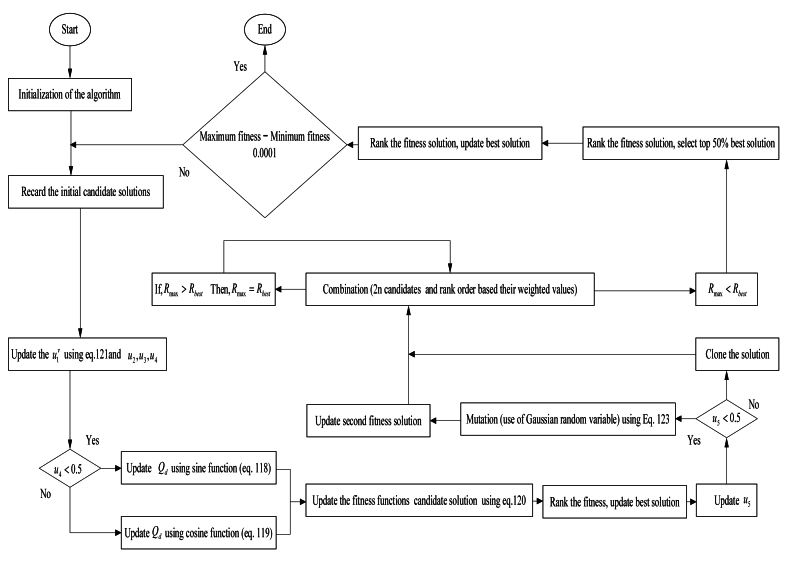
Flow chart of the EPSCA.

The objective functions of each folk are identified, and if $R_{\max}$ <$R_{best}$, then the best colonial solution and the best mutation process outcomes (2*m* possibilities) are combined. Thereafter, all members of the population $y_{l}$ and its offspring, $g_{k+m}$, are merged. These folks are then graded from highest to the lowest considering respective weight as its objective function. The first *m* individuals having larger weight were identified along with corresponding objective functions for the subsequent generation. Subsequently, because there is no difference between $R_{\max}$ and $R_{\min}$, the search is terminated. The method will restart from Equation (120) if the quest is not halted somehow. Update the best answer $y_{best}$ in the event that $R_{\max}$ >$R_{best}$. $R_{\max}$ = $G_{best}$. The process will then resume at the combination phase. Fig. 8 illustrates the flow diagram for the EPSCA.

The SCA can facilitate initial and subsequent exploration and exploitation of a parameter space, correspondingly. It differs from several other algorithms because of this feature. In investigating and reaping the benefits of the eigenvalue analysis space to identify maxima or minima, the SCA optimisation model offers the best outcome. Several SCA-related restrictions are on the lookup exploitation methodology for rapid convergence. SCA optimisation approach is time consuming for a high-quality solution and challenging to achieve consistently for a system of many parameter optimisations. Performance can be boosted by a SCA solution's convergence rate and resolution constancy. An upgraded version of SCA, the hybrid EPSCA, can optimise the damping controller in SMIB and multimachine systems whilst offering outcome efficacy though the result reliability and convergence rate [76].

## 3 Results

This part describes the results and discussions of the comparative analysis PIDF, PID and PI controllers using EPSCA optimisation technique based on multi-objective function approach for oscillation stability enhancement in SMIB system. Three series of cases using EPSCA optimisation approach to optimise PIDF, PID and PI damping controllers of SMIB system are simulated. All cases were conducted with TCSC PIDF, TCSC PID and TCSC PI in MATLAB environment. Table 2 shows the three different loading circumstances under four PIDF parameters, namely, N, $K_{P}$, $K_{D}$ and $K_{I}$, are optimised until the objective function's highest value has been reached. Similarly, the three PID controller parameters, namely, $K_{P}$, $K_{D}$ and $K_{I}$, were optimised under three distinct loading circumstances until the highest value of the objective function was acquired. Also, the two PI controller parameters, namely, $K_{P}$ and $K_{I}$, were optimised under three distinct loading circumstances until the highest value of the objective function was acquired.

**Table 2:** Instances involving the loading circumstances [76].

Instances	Loading Circumstance (P, Q)	
	Active Load P. (p. u)	Reactive Load Q (p. u)
Instance A-1	P = 0.50	Q = 0.3155
Instance A-2	P = 0.475	Q = 0.3172
Instance A-3	P = 0.40	Q = 0.3219

The following four methods are addressed:

1. SMIB network includes an EPSCA-optimised TCSC-PIDF controller (PIDF-EPSCA).
2. SMIB network includes an EPSCA-optimised TCSC-PID controller (PID-EPSCA).
3. SMIB network includes an EPSCA-optimised TCSC-PI controller (PI-EPSCA).
4. SMIB network includes an un-optimised TCSC-PIDF controller (PIDF-U).

**Fig. 9:**
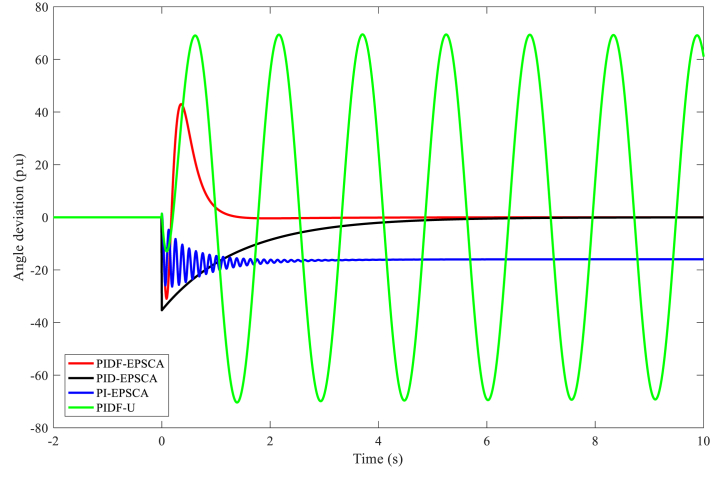
PIDF-EPSCA, PID-EPSCA, PI-EPSCA, and PIDF-U methodologies' responses to angle deviation for Instance A-1.

In juxtaposition to each other, Fig. 9 displays the outputs of the angle deviation throughout the phase plane for Instance A-1 using the PIDF-EPSCA, PID-EPSCA, PI-EPSCA and PIDF-U ways. As contrasted with the different methods, the PIDF-EPSCA system seemed to have the best damping quality; the least oscillation rate, including an overshoot of 43 p.u.; and the lowest damping time, which was around 1.35 s. PID-EPSCA followed with damped oscillations (overshoot 36 p.u) before 8.2 s, and then PI-EPSCA and PIDF-U took longer damping time. To reach the outcomes, these methods needed 8–11 iterations. The suggested approach delivered the desired function's best amount with more computing efficiency.

**Fig. 10:**
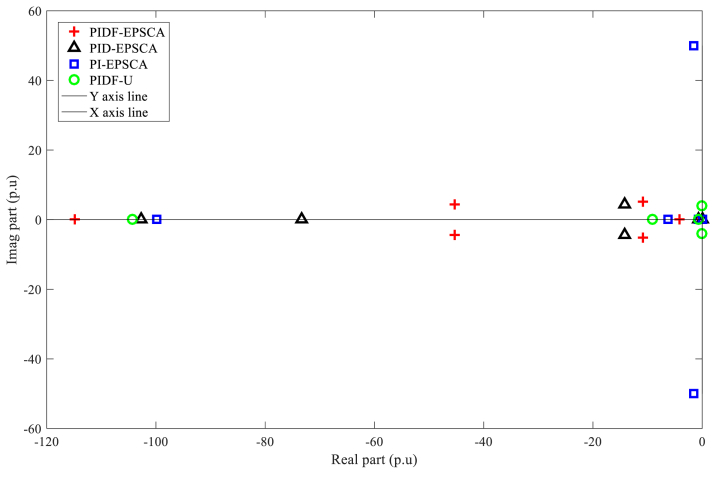
PIDF-EPSCA, PID-EPSCA, PI-EPSCA, and PIDF-U methodologies' Eigenvalues dispersion for Instance A-1.

Fig. 10 demonstrates the stable state of all units for Instance A-1 by revealing the location of all eigenvalues reside on the negative side of imaginary s-plane. In contrary to the other procedures, the eigenvalues of PIDF-EPSCA were displaced more toward the real axis of the s-plane and are localised on the left-most imaginary axis of s-plane. Meanwhile, one pair of eigenvalues of PI-EPSCA and PIDF-U systems is located at the farthest position from the real axis and close to the imaginary axis line in s-plane, thereby justifying that the technique is the worst amongst the rest. As a result, PIDF-EPSCA system provides the best solution for SMIB system stability enhancement as compared with other systems.

**Fig. 11:**
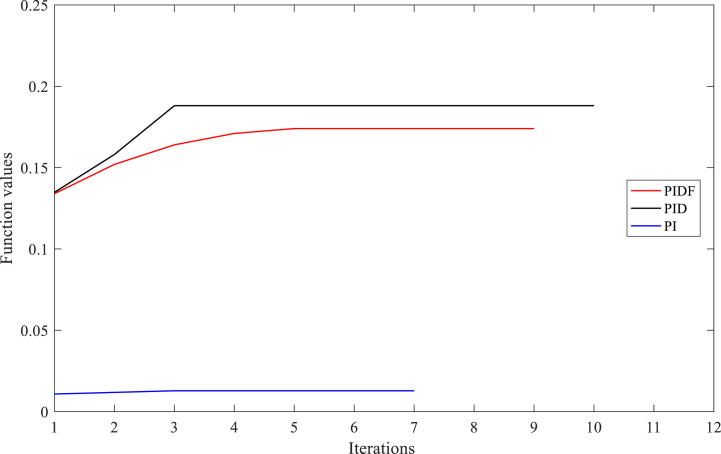
PIDF, PID, and PI methodologies' Convergence curves for Instance A-1.

Table 3 exhibits the optimised parameters of the PIDF-EPSCA controller (*N*, $K_{P}$, $K_{D}$ and $K_{I}$), PID-EPSCA controller ($K_{P}$, $K_{D}$ and $K_{I}$) and PI-EPSCA controller ($K_{P}$,and $K_{I}$), that are modified by the EPSCA optimisation technique with multi objective function as maximisation for Instance A-1. The proposed method achieved the maximum objective value, which is 0.1740. The recommended PIDF-EPSCA system takes 1.35 s to damp the low-frequency modes of oscillation (overshoot of 43 p.u) after the simulation is started with computational burden of 1.0477 s. It takes 8.2 s and longer time for the PID-EPSCA and PI-EPSCA systems to damp the low frequency modes of oscillations, respectively. For these systems to reach their solutions, 8–11 iterations (convergence curves in Fig. 11) and 0.9261 s, 0.6216 s as computational burden, were required. The low-frequency modes of oscillations with overshoots of 36 p.u, 27 p.u, and 70 p.u are dampened by the PID-EPSCA, PI-EPSCA and PIDF-U systems, respectively. This demonstrates how the recommended EPSCA methodology will stabilise the signal inside a SMIB system more effectively and regulate the TCSC controller with an optimum objective function.

**Table 3:** PIDF-EPSCA, PID-EPSCA, PI-EPSCA and PIDF-U methodologies for Instance A-1 were assessed [76].

Type	PIDF-EPSCA	PID-EPSCA	PI-EPSCA	PIDF-U
Parameters Optimised	$K_{P}$ = 0.024275	$K_{P}$ = 0.8311218	$K_{P}$ = 0.9908680	$K_{P}$ = 0.09
	$K_{D}$ = 0.000531	$K_{D}$ = 0.0982439	$K_{I}$ = 0.1357540	$K_{D}$ = 0.0002
	$K_{I}$ = 0.111213	$K_{I}$ = 0.9613662		$K_{I}$ = 0.7
	*N* = 122.1625			*N* = 120
Objective function value (p.u)	0.1740	0.1880	0.01281	NA
Number of iterations	9	11	8	NA
Settling time	1.35	8.2	>10	>10
Computational burden (s)	1.0477	0.9261	0.6216	NA
Overshoot (p.u)	43	36	27	70

**Fig. 12:**
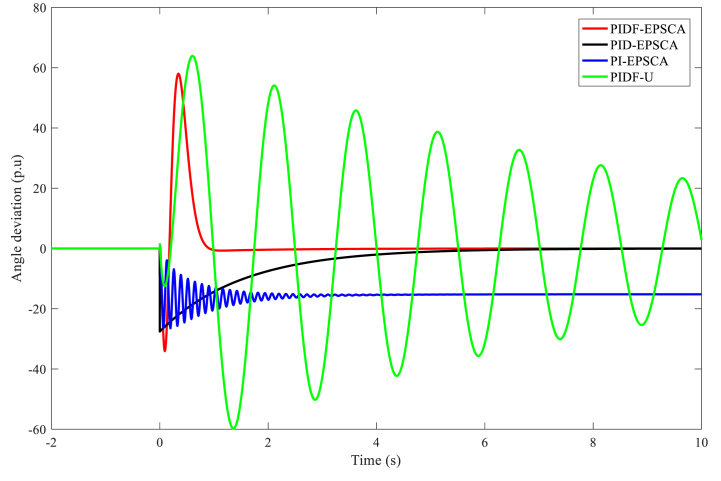
PIDF-EPSCA, PID-EPSCA, PI-EPSCA, and PIDF-U methodologies' responses to angle deviation for Instance A-2.

In comparison, the results of the PIDF-EPSCA, PID-EPSCA, PI-EPSCA and PIDF-U methods for Instance A-2 are shown in Fig. 12. In relation to the other techniques, the PIDF-EPSCA system had the least oscillation rate, the amplitude of oscillation modes of overshoot 58 p.u, and damping time, all of which were about 1.1 s with computational burden 1.1947 s (in Table 4). Before 7.6 s, PID-EPSCA damped the oscillation of overshoot 27 p.u, followed by PI-EPSCA with overshoot 26 p.u, which required a longer damping period. To reach the solutions, these systems needed 8–13 iterations and computational burden 0.8959 s and 0.6421 s (Table 4), respectively. The suggested approach generated the objective function's maximum value with more computing efficiency.

**Table 4:** PIDF-EPSCA, PID-EPSCA, PI-EPSCA and PIDF-U methodologies for Instance A-2 were assessed [76].

Type	PIDF-EPSCA	PID-EPSCA	PI-EPSCA	PIDF-U
Parameters Optimised	$K_{P}$ = 0.020772	$K_{P}$ = 0.5133657	$K_{P}$ = 0.8291906	$K_{P}$ = 0.09
	$K_{D}$ = 0.000686	$K_{D}$ = 0.0865388	$K_{I}$ = 0.05676812	$K_{D}$ = 0.0002
	$K_{I}$ = 0.0892724	$K_{I}$ = 0.20419595		$K_{I}$ = 0.7
	*N* = 142.90544			*N* = 120
Objective function value (p.u)	0.1895	0.1585	0.01266	NA
Number of iterations	13	11	8	NA
Settling time (s)	1.1	7.6	>10	>10
Computational burden (s)	1.1947	0.8959	0.6421	NA
Overshoot (p.u)	58	27	26	64

**Fig. 13:**
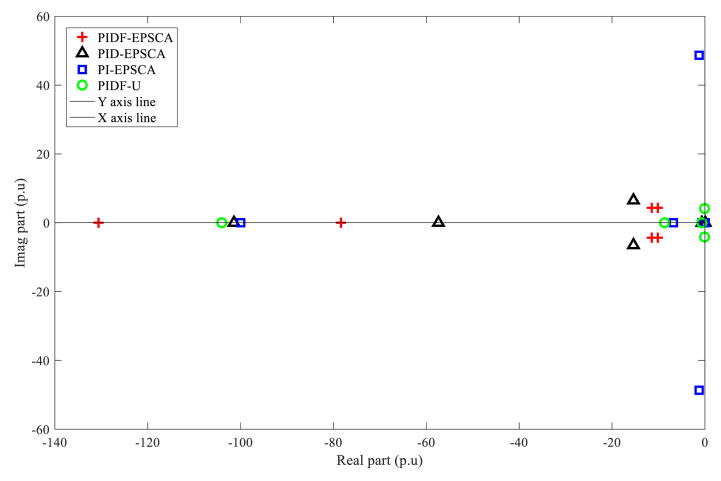
PIDF-EPSCA, PID-EPSCA, PI-EPSCA and PIDF-U methodologies' eigenvalue dispersion for Instance A-2.

Fig. 13 demonstrates that for Instance A-2, all eigenvalues reside on the complex s-negative plane's side, thereby confirming that all systems are stable. In comparison with the other approaches, the eigenvalues of PIDF-EPSCA are displaced toward the horizontal line of the s-plane and are located on the left-most imaginary axis of the imaginary s-plane. In the poorest performance in locating eigenvalues in D-shaped area by the other techniques, the PI-EPSCA system's one pair of eigenvalues is situated at the position that is the furthest from the horizontal line and near-at-hand imaginary axis line in s-plane. As a result, when compared with the two other systems, the PIDF-EPSCA system offers the most favourable approach that represents improved SMIB system stability.

**Fig. 14:**
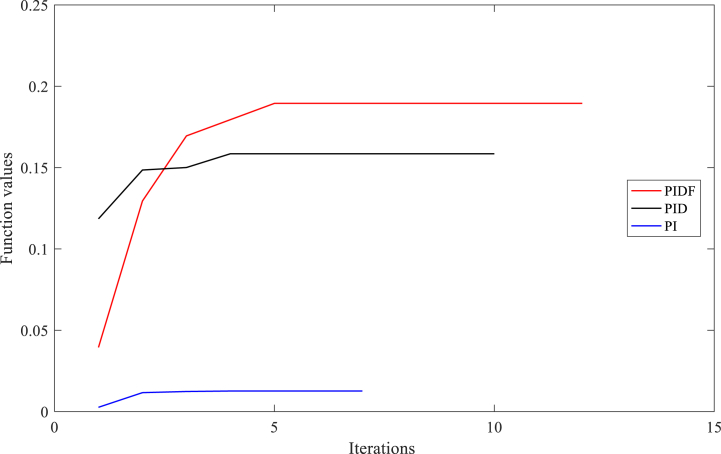
PIDF, PID, and PI methodologies' Convergence curves for Instance A-2.

The optimised parameters for the PIDF-EPSCA controller (e.g. *N*, $K_{P}$, $K_{D}$ and $K_{I}$), the PID-EPSCA controller ($K_{P}$, $K_{D}$ and $K_{I}$), the PI-EPSCA controller ($K_{P}$ and $K_{I}$) and the un-optimised parameters of PIDF-U controller are shown in Table 4. These controllers were tuned using the EPSCA optimisation technique with the multi-objective function as maximisation for case A-2. The proposed method successfully attained the highest objective value of 0.1895. After the simulation begins, the proposed PIDF-EPSCA system takes 1.1 s to damp the low-frequency oscillation modes. The PID-EPSCA and PI-EPSCA systems require 7.6 s and longer, to damp the low-frequency oscillation modes, respectively. These systems required 8–13 iterations (convergence curves in Fig. 14) to obtain their solutions. The outcomes establish that EPSCA technique may improve signal stabilization in a SMIB system and results in appropriate selection of TCSC system parameters with based on optimum objective function.

**Fig. 15:**
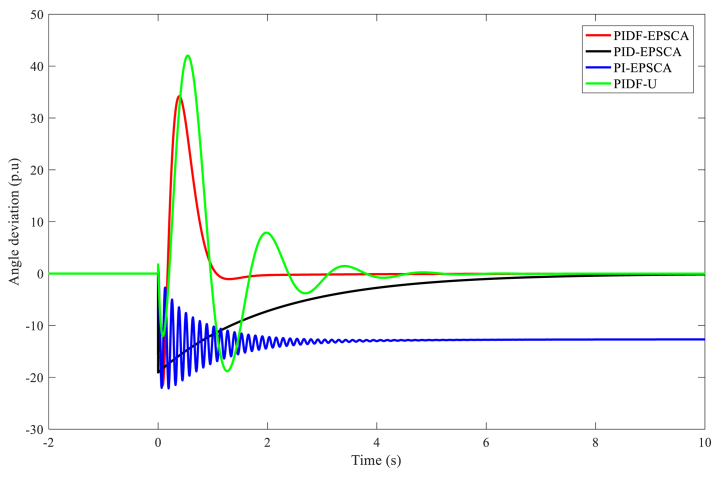
PIDF-EPSCA, PID-EPSCA, PI-EPSCA, and PIDF-U methodologies' responses to angle deviation for Instance A-3.

For Instance A-3, Fig. 15 compares the results of the angle deviation in the phase plane using the PIDF-EPSCA, PID-EPSCA, PI-EPSCA and PIDF-U approaches. In comparison to the other techniques, the PIDF-EPSCA system exhibited the least oscillation rate, satisfactory overshoot (34 p.u), the shortest damping time (about 1.47 s), and the best damping performance. Before 9.5 s, PID-EPSCA started to dampen the oscillation with overshoot (19 p.u), PIDF-U is at third place with maximum overshoot of 43 p.u, and PI-EPSCA took longer (overshoot of 22 p.u). For these systems to reach a solution, 10–12 iterations were necessary. With satisfactory computing efficiency (1.2130 s) as compared, the suggested method delivered the objective function's maximum value.

**Fig. 16:**
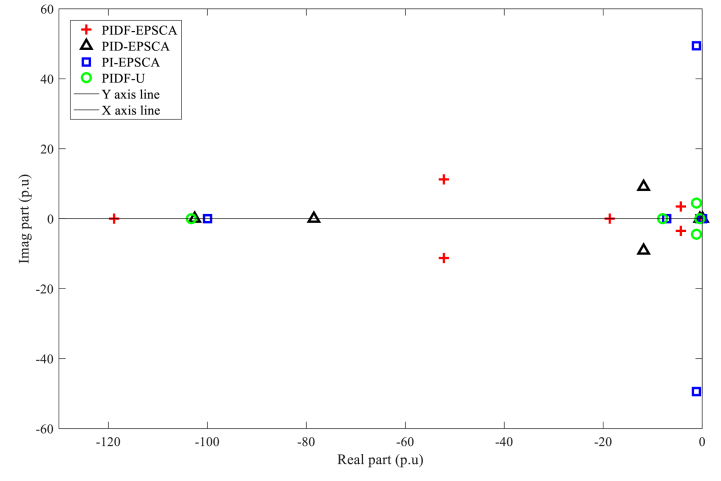
PIDF-EPSCA, PID-EPSCA, PI-EPSCA, and PIDF-U methodologies' Eigenvalues dispersion for Instance A-3.

The location of the eigenvalues for Instance A-3 is mainly associated with the negative side of the aforementioned complex s-plane, which is demonstrated in Fig. 16, thereby confirming that all systems are stable. When compared with the eigenvalues of the other approaches, those of PIDF-EPSCA are located at the leftist of the chimerical axis of the s-plane and are displaced in favour of the real axis. A pair of PI-EPSCA and PID-U system eigenvalues that are the furthest from the real axis and close to the chimerical axis line in s-plane, however, support the claim that the approach is the poorest of the bunch. The PIDF-EPSCA system delivers the best outcomes based on the validation with other models and is considered suitable to enhance the stability of the SMIB system in contrast to the three other systems.

**Fig. 17:**
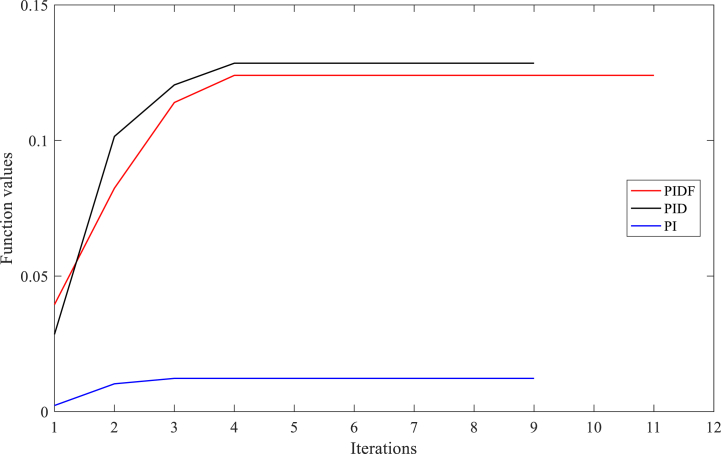
PIDF, PID, and PI methodologies' Convergence curves for Instance A-3.

Table 5 lists the optimised values for the PIDF-EPSCA controller's (*N*, $K_{P}$, $K_{D}$ and $K_{I}$), PID-EPSCA controller's ($K_{P}$, $K_{D}$ and $K_{I}$), PI-EPSCA controller's ($K_{P}$ and $K_{I}$) parameters and un-optimised PIDF-U system parameters. These parameters were tuned using the EPSCA optimisation technique with the multi-objective function as maximisation for Case C-3. The greatest objective value, or 0.1240, was attained by the suggested procedure. The low-frequency oscillations are dampened by the suggested PIDF-EPSCA system after the simulation has begun, which takes 1.47 s. The PID-EPSCA and PI-EPSCA systems dampen the low-frequency oscillations in 9.5 s and longer, with computational burden 0.8442 s and 0.7805 s, respectively. A total of 10–12 iterations (convergence curves in Fig. 17) were necessary for these systems to converge at their solutions. The PIDF-U system dampens the low-frequency oscillations with overshoot of 43 p.u in more than 10 s. This finding depicts how, in a SMIB system, the recommended EPSCA approach may be more effective at stabilising the signal and configuring the TCSC controller parameters with such an optimum objective function.

**Table 5:** PIDF-EPSCA, PID-EPSCA, PI-EPSCA, and PIDF-U methodologies for Instance A-3 were assessed [76].

Type	PIDF-EPSCA	PID-EPSCA	PI-EPSCA	PIDF-U
Parameters Optimised	$K_{P}$ = 0.011841	$K_{P}$ = 0.4810198	$K_{P}$ = 0.910891	$K_{P}$ = 0.09
	$K_{D}$ = 0.000825	$K_{D}$ = 0.1182455	$K_{I}$ = 0.197537	$K_{D}$ = 0.0002
	$K_{I}$ = 0.004116	$K_{I}$ = 0.52806133		$K_{I}$ = 0.7
	N = 77.39919			N = 120
Objective function value (p.u)	0.1240	0.1285	0.01225	NA
Number of iterations	12	10	10	NA
Settling time (s)	1.47	9.5	>10	>10
Computational burden (s)	1.2130	0.8442	0.7805	NA
Overshoot (p.u)	34	19	22	43

## 4 Discussion

The development of a reliable damping controller to reduce low frequency oscillation in power systems is the primary goal of this research. This is accomplished by creating a mathematical model of the PIDF controller and modelling it using an optimisation method, an objective function, and a damping scheme (TCSC) based on FACTS. In contrast to PID, PI, and PIDF-U controllers, the suggested EPSCA dampens low frequency oscillations by optimizing all of the PIDF controller's parameters through the use of a recommended multi-objective function, linearising the nonlinear PIDF-TCSC framework, and an eigenvalue analysis technique. Performance was evaluated under three distinct loading instances. The features of the SCA and EP procedures are combined in the hybrid EPSCA approach.

In the EPSCA approach flow chart, Fig. 8 displays the contributions made by the SCA and EP. Exploration and exploitation in a search space are achievable with the initial and subsequent SCA iterations, accordingly. This attribute sets it apart from various other algorithms. SCA optimisation usually produces superior outcomes when searching the search space and locating maxima or minima utilising the eigenvalue analysis. The sole limitation with SCA is on the search exploitation approach for fast convergence. The SCA optimisation approach has limitations with consistent results and takes a long time to deliver good results for systems that require a large number of parameters for optimisation. The SCA's inadequate pace of convergence is the cause of its inconsistent solution. Hybrid EPSCA, a modified version of SCA, is the solution to this SCA problem. It takes advantage of the best aspects of EP, possesses an outstanding convergence rate, and achieves solution performance by boosting its convergence rate and result accuracy.

The proposed optimisation approach delivers a system that boosts the damping abilities by using a suggested multi objective function, as demonstrated by the angle deviation responses and eigenvalue distributions of the TCSC-PIDF controller system using the EPSCA technique under various loading instance. The findings indicate, relative to traditional techniques, the suggested PIDF-EPSCA approach is potentially able to stabilise the signal in a SMIB power system and optimise the TCSC-PIDF controller settings with maximum values for the objective function, as well as a high rate of convergence and settling time. PIDF controllers have been illustrated for damping power system oscillations in interconnected power systems. However, PIDF controllers are challenging task to model large interconnected power system because of their high-order derivative terms and optimise large parameters for multimachine system to asses the stability.

## 5 Conclusions

The main research objective considered relates with the development of a dependable damping controller. The proposed damping controller is developed to ensure the reliability of a SMIB system from generating the low-frequency oscillations within power supply. By creating a design of the PIDF controller, establishing a desirable objective function and creating an effective EPSCA optimisation procedure, this research created a resilient damping controller. To accomplish the objective, a new controller (PIDF) was developed using D-shaped objective function and hybrid EPSCA optimisation technique for a SMIB system. FACTS TCSC PIDF controller was described to ensure the stability of the power system. Three different models of SMIB system were studied to analyse the designed controllers in terms of stability. The enhanced system damping of these models were evaluated based on settling time and overshoot of different low-frequency oscillation modes obtained from simulations. Comparing MO-EPSCA based PIDF damping controller performance to other controller's techniques, it demonstrated a significant enhancement in overshoots and settling times for low-frequency oscillation modes (TCSC-PID, TCSC-PI. and PIDF-U). EPSCA PIDF design controllers are effective to damp all inter-area modes of oscillations. In addition, EPSCA PIDF-based design controllers may provide efficient damping performance in SMIB power systems. Thus, the implementation of the designed PIDF controller efficiently dampens the low-frequency oscillations in the SMIB system. Recommendation for future work, to establish robust damping controller for the multimachine large power system by implementing a novel index, PIDF modelling, and EPSCA for both PSS and FACTS different schemes. Furthermore, to implement a new index, PIDF controller, and EPSCA to design coordination control of PSS and FACTS robust damping controllers for the multimachine large power system to enhance damping performance.

## CRediT authorship contribution statement

**Abdul Waheed Khawaja:** Writing – review & editing, Writing – original draft, Visualization, Validation, Software, Resources, Project administration, Methodology, Investigation, Formal analysis, Data curation. **Nor Azwan Mohamed Kamari:** Writing – review & editing, Visualization, Validation, Supervision, Software, Resources, Project administration, Funding acquisition. **Muhammad Ammirrul Atiqi Mohd Zainuri:** Writing – review & editing, Supervision, Project administration. **Syahirah Abd Halim:** Writing – review & editing, Supervision. **Mohd Asyraf Zulkifley:** Writing – review & editing, Project administration. **Shaheer Ansari:** Writing – review & editing, Validation, Project administration. **Abdul Sattar Malik:** Writing – review & editing, Supervision.

## Declaration of competing interest

I wish to submit an original research article entitled ‘Angle Stability Improvement Using Optimised Proportional Integral Derivative with Filter Controller’ for consideration by ***Heliyon***. I confirm that this work is original and has not been published elsewhere, nor is it currently under consideration for publication elsewhere. The authors declare no conflict of interest.
